# Use of cultivation-dependent and -independent techniques to assess contamination of central venous catheters: a pilot study

**DOI:** 10.1186/1472-6890-8-10

**Published:** 2008-10-28

**Authors:** Mette KS Larsen, Trine R Thomsen, Claus Moser, Niels Høiby, Per H Nielsen

**Affiliations:** 1 Department of Biotechnology, Chemistry, and Environmental Engineering, Aalborg University, Sohngaardsholmsvej 49, DK-9000 Aalborg, Denmark; 2 The Danish Technological Institute, Chemistry and Water Technology, Kongsvang Allé 29, DK-8000 Århus C, Denmark; 3 Department of Clinical Microbiology, Rigshospitalet, University of Copenhagen, Juliane Maries Vej 22, DK 2100 Copenhagen, Denmark

## Abstract

**Background:**

Catheters are the most common cause of nosocomial infections and are associated with increased risk of mortality, length of hospital stay and cost. Prevention of infections and fast and correct diagnosis is highly important.

**Methods:**

In this study traditional semiquantitative culture-dependent methods for diagnosis of bacteria involved in central venous catheter-related infections as described by Maki were compared with the following culture-independent molecular biological methods: Clone libraries, denaturant gradient gel electrophoresis, phylogeny and fluorescence in situ hybridization.

**Results:**

In accordance with previous studies, the cultivation of central venous catheters from 18 patients revealed that *S. epidermidis *and other coagulase-negative staphylococci were most abundant and that a few other microorganisms such as *P. aeruginosa *and *K. pneumoniae *occasionally were found on the catheters. The molecular analysis using clone libraries and sequencing, denaturant gradient gel electrophoresis and sequencing provided several important results. The species found by cultivation were confirmed by molecular methods. However, many other bacteria belonging to the phyla *Proteobacteria, Firmicutes, Actinobacteria *and *Bacteroidetes *were also found, stressing that only a minor portion of the species present were found by cultivation. Some of these bacteria are known to be pathogens, some have not before been described in relation to human health, and some were not closely related to known pathogens and may represent new pathogenic species. Furthermore, there was a clear difference between the bacterial species found in biofilm on the external (exluminal) and internal (luminal) side of the central venous catheter, which can not be detected by Maki's method. Polymicrobial biofilms were observed on most of the catheters and were much more common than the cultivation-dependent methods indicated.

**Conclusion:**

The results show that diagnosis based on molecular methods improves the detection of microorganisms involved in central catheter-related infections. The importance of these microorganisms needs to be investigated further, also in relation to contamination risk from improper catheter handling, as only in vivo contaminants are of interest. This information can be used for development of fast and more reliable diagnostic tools, which can be used in combination with traditional methods.

## Background

Intravenous catheters are a commonly used medical device, but their use is often associated with increased risks for infections [1]. In Europe more than 60% of all nosocomial infections in European hospitals are associated with a catheter, the majority being central venous catheters (CVC) [2].

Diagnosis of CVC-associated infection traditionally relies on clinical features, quantitative cultivation, Gram staining, and acridine-orange leucocyte cytospin test of blood samples [3-5] or on methods that can only be applied following CVC removal [6]. The international reference diagnostic method is Maki's semi-quantitative method (1973), which is based on the removal of the catheter and rolling the distal tip back and forth on an agar plate [3,7]. Fourteen colony-forming units (CFU) define an insertion site infection [7]. There are, however, several problems with these methods for diagnosis. It takes at least 2 days to obtain a result and as only the catheter tip is rolled back and forth on the agar plate, bacteria further up the catheter and on the internal site are not included in the analysis. Furthermore, it has been shown that many catheters appear sterile (are culture negative) after removal in cases where there were signs of infections [8,9]. These observations suggest additional diagnostic methods are necessary in cases where CVC-related infections are suspected.

The problem with Maki's method, and other diagnostic methods based on cultivation, is that they do not always detect all microorganisms involved in the infections due to the use of selective growth media and the poor viability of some microorganisms on these media [10-12]. This may be due to antibiotic treatment but more likely because the microorganisms grow in biofilms. Biofilm-forming bacteria behave quite differently from planktonic cells and many cultivation-based diagnostic tools are based on planktonic organisms [13]. A recent European study of intravascular catheter-related infections found that the microorganisms most often isolated from intravascular catheters were coagulase-negative staphylococci (30–51.5%), followed by *Candida *spp., *Staphylococcus aureus, Enterococcus *spp. and *Pseudomonas *spp. [3,14,15]. *Acinetobacter *spp. [3,16], *Enterobacter *spp. [3], *Klebsiella pneumonia *[15] (all *Gammaproteobacteria*) and other bacteria have been identified less frequently. However, as described above, many biofilm-forming bacteria are poorly detected [11], so potentially several other bacteria may be involved in the CVC-related infection without being detected by traditional culture-based diagnostics. This means that delayed or even incorrect diagnosis can occur thus increasing the risk of relapse or systemic infection, resulting in pain and discomfort, a less satisfactory outcome for the patient, technical difficulties for the health care provider and significant costs for the health service [17]. These problems demonstrate the need for new diagnostic methods, independent of cultivation. Molecular techniques have been used successfully for identification of bacteria in other systems e.g. [18-20], including some medico-related [6,21,22], but have only been used for diagnosis of microbial infections in rather few cases on individual CVCs using broad range 16S rRNA gene sequencing (see e.g. [23,24]).

The aim of this study was to compare the traditional, culture-dependent methods for the diagnosis of bacteria involved in CVC-infections with several culture-independent molecular methods to improve the diagnosis of CVC-related infections.

## Methods

### Cultivation

All unselected central venous catheters sent to the Department for Clinical Microbiology, Copenhagen, during a 3 months period were included in this study. All catheters were generally handled under aseptic conditions. The catheter tips were analysed by Maki's semi-quantitative method [7], where the catheter tips were rolled back and forth on blood agar and incubated at 37°C and atmospheric air for 48 hours. Bacteria were further identified using the Api kit (Biomerieux) of automatic systems for identification, analysed on a ATB expression 1550 Vitek Systems (Biomerieux). The cultivation and identification was always performed by same trained individuals.

For blood culturing the BACTEC 9240 system was used (BACTEC, Becton Dickinson, Sparks, Maryland, USA). A minimum of two culture vials per patient, one aerobic and one anaerobic was filled directly with blood according to the manufacturers instruction. Growth of microorganisms are detected by microorganisms releasing CO_2 _modulating the fluorescence level in the sensor of the vials, which is subsequently detected by the instrument. Cultures were continued for up to one week. If detected positive, one mL from the vial was aliqouted aseptically for light microscopy (direct unstained and after Gram stain) and for culturing on a variety of agar plates (Statens Seruminstitut, Copenhagen, Denmark) for growth requirements and further identified using the Api kit of automatic systems for identification, analysed on a ATB expression 1550 Vitek Systems. The cultivation and identification was always performed by same trained individuals.

### Isolation of biofilm and DNA extraction

The internal site of the catheters was flushed with approximately 10 mL DNA buffer (0.5 M Tris/HCl, 0.5 M NaCl and 5 mM EDTA) and the biofilm was collected in a tube (samples called internal/I). Biofilm from the external site was removed with a scalpel and transferred to DNA buffer (samples called external/E). The catheter was then cut open and biofilm from the internal site was removed and transferred to the DNA buffer used to flush the internal site (internal samples/I). For some catheters it was not possible to separate biofilm from the internal from the external site and a separate clone library was constructed (mixed samples/M). The DNA extraction were performed using the FastDNA^® ^SPIN kit for soil from QBiogene. The samples were centrifuged at 13,000 rpm at 10 min and the supernatant was removed so there were approximately 100–150 μL sample left. The pellet was resuspended in the remaining supernatant and transferred to the Lysing Matrix E tube and DNA extraction was performed according to the protocol for the FastDNA^® ^SPIN kit for soil. As negative control a new catheter was rolled back and forth on blood agar and flushed and scraped as mentioned above.

### 16S rRNA gene amplification

The 16S rRNA genes were amplified by polymerase chain reaction (PCR) using *Taq *DNA polymerase with primers 8F (616V; [25]) and 1390R [26] targeting conserved domains. The samples were amplified according to [20] (using ANME primers, various numbers of cycles). Amplification of samples for DGGE were done using primer 341F-GC [27] and primer 907R [26]. Negative controls including water and PCR mix were included for every five samples and were always negative indicating there was no contamination of the reagents. Stringent procedures were employed to avoid contamination, e.g. by using a PCR cabinet with UV light and all DNA handling was done with aerosol filter pipette tips to avoid cross contamination.

### Denaturant gradient gel electrophoresis (DGGE)

DGGE analyses of the amplicons were performed on 8% polyacrylamide gels containing denaturant gradients of 20–70%. Electrophoresis was performed in 1 × TAE buffer at 100 V overnight using the D-GENE™ gel system (Biorad) and the gels were stained with Sybr Gold solution. The most intensive DGGE-bands were excised and prepared for sequencing. The excised bands were re-amplified with PCR and the PCR products were thereafter purified with NucleoSpin Extract II Machery Nagel. The similarities of the sequences from reamplified and cut DGGE bands (approximately 550 bp) were analyzed using the NCBI Blast search tool at .

### Cloning, sequencing and phylogenetic analysis

The amplified 16S rRNA gene products were purified with a Qiaquick PCR purification kit (Qiagen). Cloning was performed using a TOPO TA Cloning^® ^kit for sequencing (Invitrogen life technologies). Plasmids were purified using a Fastplasmid mini kit (Eppendorf). Purified plasmids were amplified using M13 primers to test for inserts with correct length. The clones were sequenced using a MegaBase 100 DNA sequencing system (Amersham pharmacia). Similarity searches of the retrieved 16S rRNA gene sequences against sequences deposited in publicly accessible databases were performed using the NCBI Blast search tool at . All clones were initially sequenced using the forward primer 8F, and based on the results; they were grouped according to the accession number of the closest relative in the NCBI database. Randomly selected members of each group were sequenced using the reverse primers 1390R and 907R to gain consensus sequences representing each group (almost full length ~1350 bp). All the clones with the closest relative being an uncultured bacterium were also sequenced using the reverse primers. Operational taxonomic units (OTUs) were identified using search results of at least 97% similarity. Checks for chimeric sequences were conducted using the CHECK_CHIMERA program from Ribosomal Database Project  and the program BELLEROPHON [28].

The ARB software was used for phylogenetic analysis . The sequences were aligned using the FastAligner, and subsequent manual refinement. Unambiguously aligned sequences were analysed using the distance, parsimony, and maximum likelihood approaches with default settings. Additionally, the *Bacteria *sequence conservation filter of the ssu_jan04_corr_opt ARB database (available at ) was applied. Phylogenetic trees were constructed using the consensus sequences representing the different groups of bacteria, then the partial sequences were added to the existing consensus trees by the "add species to existing tree" function in the ARB software. Prior a filter was made to define which positions to be used in adding the partial sequences (data not shown). Generally, the results obtained by NCBI Blast Search corresponded well to the positions in the phylogenetic tree. The coverage ratio (C) for each of the clone libraries were calculated with *C *= (1 - (*n*1·*N*^-1^))·100%, where n1 is the number of OTUs containing only one sequence and N is the total number of clones analysed [18].

### Fluorescence in situ hybridization (FISH)

The FISH procedure was performed on fixed biofilm [29], and visualized with a Zeiss LSM 510 (Carl Zeiss, Oberkochen, Germany) confocal laser scanning microscope. The following oligonucleotide probes were used: EUB338, EUB338-II, and EUB338-III, called EUBmix (all *Bacteria*; [30,31]), and GAM42a (*Gammaproteobacteria*; [32]). Oligonucleotides were 5'-labelled with 5(6)-carboxyfluorescein-*N*-hydroxy-succinimide ester (FLUOS) or with sulphoindocyanine dyes (Cy3) (Thermo Hybaid, Germany).

#### Nucleotide accession numbers

GenBank accession numbers for 16S rRNA gene consensus sequences determined in this study are EU160495–EU160582.

## Results

### Catheter samples

Eighteen central venous catheters were collected from seven different wards at Copenhagen University Hospital, Denmark (Table 1). Suspicion of infection was raised in cases of blood cultures with low-level pathogenic bacteria like coagulase negative staphylococci (especially if recurrent), positive blood cultures without another obvious focus of infection or cases with signs of infection at insertion site of the CVC. Finally, some CVCs were sent for culturing as routine, even without suspicion of infection.

**Table 1:** Information on catheters

Catheter number	Duration of insertion	Change of catheter	Suspicion of infection	Blood cultivation	Antibiotic treatment
1	9 days	No	No	Not taken	MEM
2	3 months	No	No	Not taken	CIP, FLC, sulfotrime
3	2 months	No	Yes	Coagulase-negative staphylococci 2 weeks before, *S. aureus *in swab	MEM, diclocile, VAN
4	14 days	No	No	Not taken	CXM
5	2–3 weeks	?	Yes	*E. faecium *2 days earlier	MEM, CIP, metronidazole, FLC
6	3 weeks	Yes	Yes	*Streptococci *6 days earlier	VAN, MEM, CIP, fluconazol
7	17 days	Yes	?	Not taken	MEM, GEN, VAN
8	No data available			*Lactococcus lactis *2 days earlier	
9	No data available			Not taken	
10	2–3 weeks	?	Yes	*E. faecium *2 days earlier	Caspofungin
11	8 days	Yes	Yes	*E. faecium *and coagulase-negative staphylococci 2 days earlier. Fungi 1 day later	CIP, MEM, fucidine, metronidazole
12	2 weeks	?	Yes	*Corynebacterium jeikeium *5 days earlier and in swab	VAN, GEN, FLC, PIP, tazabactam, VRC
13	≥ 4 weeks	Yes	No	Coagulase-negative staphylococci 8 days later	MEM, CIP, metronidazole, caspofungin
14	10 days	Yes, several times	Yes	*S. aureus *16 days earlier and fungi 7 and 11 days earlier	MEM, CIP, VAN, fucidine, amphotericinB
15	5 days	No	Yes	*P. aeruginosa *4 days earlier and in swab	CIP, MEM
16	17 days	Yes	No	Not taken	MEM, CIP, GEN, colistine
17	5 days	No	Yes	Not taken	CIP, MEM
18	No data available			Not taken	

Data about the catheters including insertion time, catheter changes, suspicion of infection, blood cultivation, and the antibiotic treatment the patient retrieved during catheterization.

For 3 patients it was not possible to receive any data at all about the catheterization. From the patients with available information, the time of catheterization ranged from 5 days to 3 months. In 9 cases there were suspicion of infection and in 6 cases the catheter had previously been changed, and in one case the catheter was changed several times. All patients received antibiotic treatment before or during catheterization based on suspicion of infection.

### Cultivation data

Immediately after removal of the catheters from the patients, the catheter tip was analysed by Maki's semi-quantitave method (Table 2). *S. epidermidis *and other coagulase-negative staphylococci were most abundant and *P. aeruginosa, K. pneumoniae *and some fungi were detected on one catheter each. Cultivation did not detect any bacteria on seven of the catheters although there was suspicion of infections in four of these based on blood cultivation data (Table 1). Two catheters had mixed growth based on cultivation (1 and 18), and the bacteria on catheter 18 could not be identified further. Coagulase-negative staphylococci, streptococci, *E. faecium, C. jeikeium, S. aureus, P. aeruginosa *and *L. lactis *were identified in blood samples mainly before the catheters were removed.

**Table 2:** Cultivation data

Species	No. of catheters	Percent [%]	Catheter number
No growth	7	36.8	3,6,7,9,10,12,16
Coagulase-negative staphylococci	4	21.1	2,13,14,15
*S. epidermidis*	4	21.1	1,4,5,8
*P. aeruginosa*	1	5.3	17
*K. pneumoniae*	1	5.3	1
Fungi	1	5.3	11
Mixed growth	1	5.3	18

Cultivation data from the 18 CVC investigated. All except those identified as mixed growth were quantified to more than 15 CFU.

### Molecular methods

It was possible to separate the biofilm from the external and internal sites for 14 of the 18 catheters. For 4 of the catheters (sample number 9, 10, 12 and 18) this was not possible and a separate clone library was constructed based on subsamples from these mixed biofilms (called mixed/M).

#### Clone libraries

The results are presented in Table 3 and they are based on both partial and consensus sequences. All of the bacteria detected by cultivation, excepting *K. pneumoniae*, were also detected in the clone libraries, however in all cases a large number of other bacteria were also present. The most abundant clone on the external site was *S. epidermidis*, which was also found to be abundant by cultivation. Fungi were not included in the molecular analysis.

**Table 3:** Clone library data

	Bacterial species	External	Internal	Mix
*Alphaproteobacteria*	*Afipia broomeae*			3 (98%)
	*Bradyrhizobium japonicum*			5 (96–98%)
	*Bradyrhizobium *sp.			1 (96%)
*Betaproteobacteria*	*Acidovorax *sp.			3 (94–99%)
	*Alcaligenes *sp.			2 (96–98%)
	*Burkholderia cepacia*			1 (96%)
	*Burkholderia *sp.	1 (97%)		3 (98–99%)
	*Delftia tsuruhatensis*	1 (96%)		
	*Diaphorobacter *sp.			1 (98%)
	*Massilia *sp.	2 (97–98%)		
*Gammaproteobacteria*	*Acinetobacter junii*		11(90–99%)	
	*Acinetobacter *sp.		10(98–99%)	
	*Pseudomonas aeruginosa*	1 (95%)	11(94–99%)	
	*Pseudomonas *sp.		1 (97%)	
	*Serratia *sp.			2 (99%)
	*Stenotrophomonas maltophilia*	3 (98–99%)	3 (96–98%)	1 (97%)
	*Stenotrophomonas *sp.		3 (96–99%)	
*Deltaproteobacteria*	Uncultured *Deltaproteobacteria*	9 (97–99%)		
*Firmicutes*	*Enterococcus faecium*	1 (99%)	2 (98%)	
	*Enterococcus lactis*		1 (98%)	
	*Peptostreptococcus octavius*			5 (93–95%)
	*Staphylococcus epidermidis*	34 (88–99%)	16 (95–99%)	5 (96–99%)
	*Staphylococcus haemolyticus*	3 (95–98%)		
	*Staphylococcus pasteuri*		3 (91–99%)	
	*Streptococcus pneumoniae*			1 (97%)
	Uncultured *Bacillales *bacterium	1 (92%)		
*Actinobacteria*	*Corynebacterium *sp.		1 (99%)	
	*Kocuria rhizophila*	1 (99%)		
	*Micrococcus luteus*	1 (97%)		
	*Propionibacterium acnes*	2 (98%)		
Unknown	Uncultured bacterium clone 654931	2 (98–99%)		1 (99%)
	Uncultured organism clone MC060411			1 (95%)

	Total	62	62	35

Blast results of the retrieved sequences from the three clone libraries from external, internal and mixed biofilm samples. Numbers refers to abundance of the different clones and the numbers in parentheses denote the percent ID, the clones have been identified with in the Blast search.

**Figure 1:**
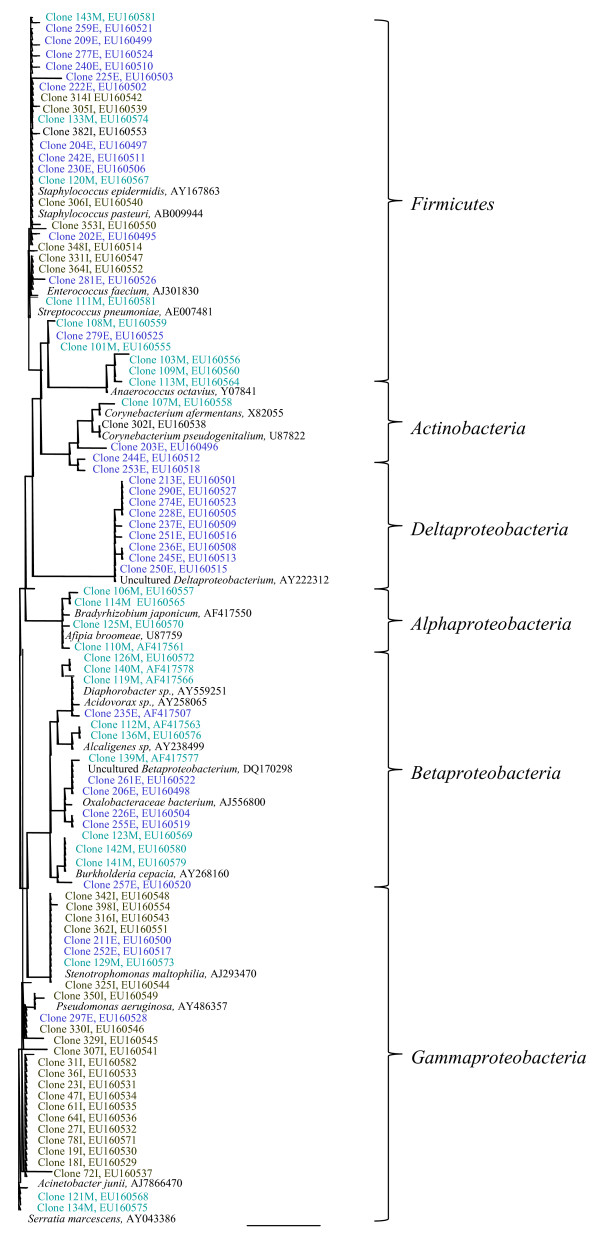
**Phylogenetic tree based on sequences obtained from the biofilm samples.** "I" after a clone number refers to a biofilm sample from the internal site, "E" refers to a biofilm sample from the external site, and "M" refers to biofilm from a mixed sample. Only consensus sequences are shown in the tree. The scale bar of 0.1 corresponds to 10% estimated sequence deviation. *Gammaproteobacteria*, the *Firmicutes *and *Actinobacteria *were present on all three sample types, while *Beta*- and *Deltaproteobacteria *were only present in samples from the external site and *Alphaproteobacteria *in mixed samples.

Further phylogenetic analysis of the sequences obtained from the 3 clone libraries and their closest relatives found by the BLAST search were performed in ARB. The methods applied generally resulted in congruent tree topologies, and the maximum likelihood tree is shown illustrating the phylogeny of the consensus sequences (Figure 1). Three phyla were represented in the clone library for the external site: *Proteobacteria (Beta*-, *Gamma*- and *Deltaproteobacteria*), the *Firmicutes*, and the *Actinobacteria*. The group with most clones in this clone library was the *Firmicutes*, dominated by the staphylococci (constituted 59.7% of the identified clones). "Uncultured *Deltaproteobacteria*" was represented with 9 sequences (constituted 14.5% of the identified clones) while a number of clones were found 1–3 times. Two sequences (number 206 and 261) were denoted "uncultured bacterium clone 654931" from the BLAST search, but as can be seen by the phylogenetic tree (Fig. 1), they seem to belong to the *Betaproteobacteria*.

The internal site was represented by 3 phyla: the *Gammaproteobacteria*, *Firmicutes*, and *Actinobacteria*. The *Gammaproteobacteria *was the biggest group with 39 sequences (62.9%), followed by the coagulase-negative staphylococci constituting 30.6% of the clone library.

The mixed sample contained *Proteobacteria *(*Alpha*-, *Beta*- and *Gammaproteobacteria*), *Firmicutes*, and *Actinobacteria*. *Firmicutes *accounted for the biggest group, followed by the *Alpha*- and *Betaproteobacteria*. It was the only clone library containing *Alphaproteobacteria*. The phylogenetic relationship of clone number 107 was resolved using ARB, which supported its relatedness to *Corynebacterium *sp.

The coverage ratio for the 3 clone libraries was high for the clone library of the internal site (96.8%) and for external site (90.2%), indicating that most of the organisms in the sample had been detected. The lowest coverage ratio is from the mixed sample (85.8%), which was based only on 35 sequences, compared to approximately 60 sequences for both the external and internal site.

#### Denaturant gradient gel electrophoresis (DGGE)

**Figure 2:**
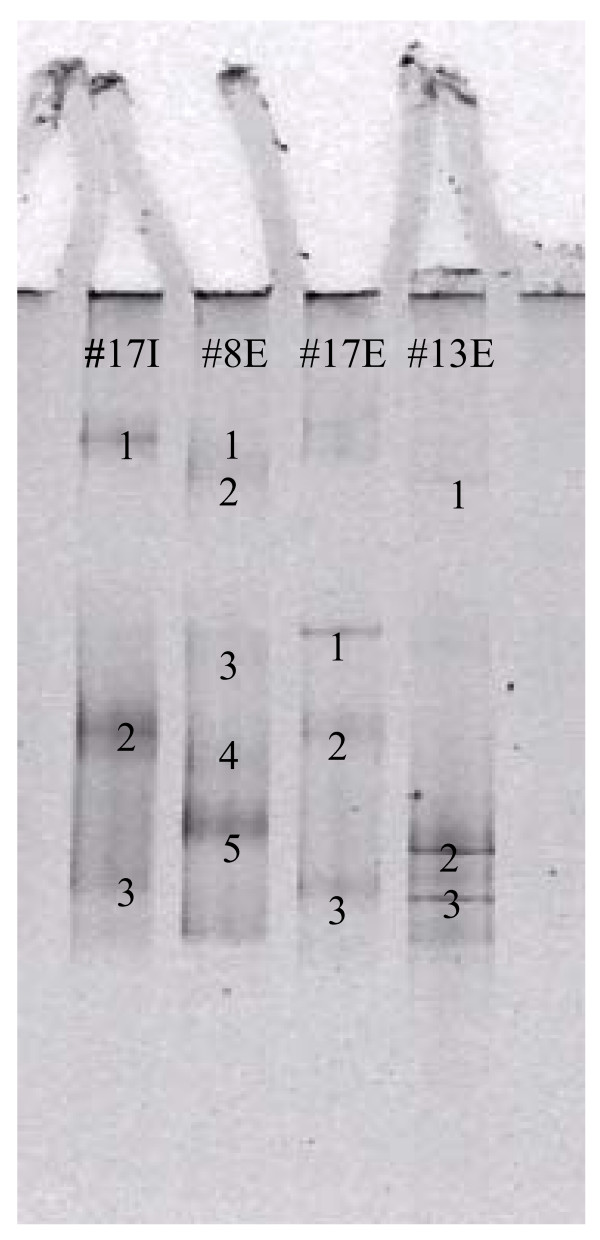
**Example of a typical DGGE gel.** The sample present in a lane is written at top of the lane. "I" refers to a biofilm sample from the internal site and "E" to a biofilm sample from the external site. The numbers denote bands which were applicable for reamplification and sequencing.

DGGE was performed on biofilm samples from the individual catheters in order to gain more information about the bacterial diversity. In most DGGE lanes several bands were present, which means that numerous species were present in the original biofilms. The number of clear bands ranged from 2 to 14. Selected bands were excised, reamplified and sequenced in order to identify the bacteria represented by that band. Figure 2 presents a representative DGGE gel, demonstrating the variety of banding patterns and the numbers represent bands applicable for reamplification and sequencing. A band on the DGGE gel is assumed to represent one bacterial species/subspecies and accordingly there seemed to be several species present in the biofilm on both the external and internal site of the individual catheters. It was possible to cut out bands from all the samples except from the internal site of catheter 16. In the majority of the samples, more than one band was applicable for reamplification and sequencing, even though some of the bands appear rather weak on the images. The presence of only one species was found in biofilm from three catheter samples using DGGE, whereas the biofilm from the other catheter samples was definitely polymicrobial. The bacteria identified using DGGE are described in Additional file 1. On many catheters the DGGE results, especially the external site, were consistent with the cultivation data. However, many other microorganisms were identified on the individual catheters and significant differences were observed between samples from internal and external site in most cases.

#### Fluorescence in situ hybridization (FISH)

The abundance of bacteria in most samples was too low for FISH detection. To illustrate the potential of the method, it was possible to locate *Gammaproteobacteria *in sample 2 by FISH with probe GAM42a (data not shown). Interestingly, the clone libraries and DGGE also detected *Gammaproteobacteria *in this sample in contrast to the cultivation data (catheter 2, Table 2).

## Discussion

The comprehensive combination of molecular methods used in this study has not been used in relation to microbial colonization of CVC previously, but has recently been used in another study related to human health on atherosclerotic lesions in patients with coronary heart disease [21]. These methods substantially improved the diagnostic outcome in the study and thus strongly support the finding in this study that the traditional culture-dependent methods can benefit from supplemental molecular diagnostics.

Five important results were obtained from the comparison of the cultivation results with the results from the molecular analysis using clone libraries, DGGE and sequencing: 1) The species found by cultivation was also found by molecular methods, 2) Many other bacteria (or, more correctly, clones or sequences) were also found stressing that only a minor part of the species present was found by cultivation, 3) Some of these bacteria described by molecular methods are not closely related to known pathogens and may represent new pathogenic species or harmless colonizers, 4) There was a clear difference between the bacterial species on the external and internal site of the CVC, 5) Polymicrobial biofilms seem much more common than the cultivation-dependent methods indicated. These results are further discussed below.

### Microorganisms on CVC as detected by molecular methods

Many different bacterial species were found using the molecular techniques and many of them have been isolated from CVCs in our study or in other studies. These include coagulase-negative staphylococci, *S. aureus, Enterococcus *spp., *Corynebacterium *spp., *Micrococcus *spp., *Burkholderia *spp., *Stenotrophomonas *spp., *Pseudomonas *spp. *Acinetobacter *spp. and *Enterobacter *spp.

Other bacterial species were found, which hitherto have not been related to CVC colonization, but are well known in relation to other infections. *Acidovorax *sp. (clones were found with 94–99% similarity to known *Acidovorax*) was initially classified as *Pseudomonas*, they are environmental bacteria, found in soil, water and as plant pathogens, but has recently been isolated from blood cultures from a human patient with sepsis, indicating that this organism has a pathogenic potential in the healthcare setting [33]. *Massilia timonia *(97–98% similarity) has been involved in human infections, e.g. wound infections [34] and also been isolated from blood cultures from an immunocompromised patient [35]. *Peptostreptococcus octavius *(93–95% similarity) is a member of the human microflora and is frequently isolated from sites of infections and comprise approximately one quarter of all isolates from anaerobic infections [36]. Both *Massilia *sp. and *P. octavius *are slowly growing and fastidious and therefore may be difficult to detect using the culture-dependent method, if the incubation time is less than 48 h and is not anaerobic. *Propionibacterium acnes *(98% similarity) is part of the normal microflora of human skin and is recognized for its role in acne vulgaris [37], but do also cause infections in patients with implantable prostheses, where it has been associated with prosthetic valve related endocarditis [38].

Lastly, some bacteria were identified that have not been related to human health before e.g. the alphaproteobacterial *Afipia*, which have been isolated from hospital water supply and is thereby present in the hospital environment [39]. The clones identified as *Afipia broomeae *had a similarity of 98%. *Bradyrhizobium *sp. has been isolated in biofilm in dental waterline tubing [40], but not found to be related to any human diseases. These are symbiotic nitrogen fixers that can be found in the roots of plants [41] and they were identified with a similarity of 96–98%. The betaproteobacterial *Delftia tsuruhatensis *(96% similarity) is a terephthalate-assimilating bacterium [42] and *Diaphorobacter *sp. (98% similarity) is a denitrifying bacterium [43] and are both isolated from activated sludge.

On seven catheters no growth were observed with the cultivation, but all catheters were represented by bands on the DGGE, which were possible to amplify and sequence, except catheter 7, where "no significant match" was achieved by sequence analysis. The results obtained by DGGE from the catheters with no growth in many cases showed bacteria, not normally isolated from central venous catheters, including *Delftia tsuruhatensis, Propionibacterium acnes *and *Afipia *sp. They were also found in the clone libraries. However, the blood cultivation data from these seven patients before the catheters were removed correlated with DGGE results in catheter number 3 and 11, but not number 6, 10 and 12. A relatively big group from the external site in the clone library was the "uncultured *Deltaproteobacteria*" and they were also detected in biofilm from the external site of catheter 16 using DGGE. Unfortunately, the clones could not be identified further. The *Deltaproteobacteria *includes amongst others a large group of sulfate-reducing bacteria [44].

Generally, most bacteria found by the DGGE approach were also identified in the clone library, except some uncultured organisms and some sequences related to *Wolbachia *and *Jenibacter molonis*. In contrast, many bacteria were found only in the clone library, supporting that DGGE only detects the dominating bacteria present in a sample.

So even though many of the bacteria found in the clone libraries have not previously been related to CVC-related infections, several are related to the hospital environment and may be involved in nosocomial infections. Many sequences were found where the closest relatives were uncultured organisms, of which the significance is unknown. However, many of these results were based on a relatively low similarity with known organisms, and they could either be really unknown species or be a result of PCR or sequencing errors. Therefore, the role of the detected, but relatively unknown bacteria needs to be resolved in future studies in order to investigate whether they are of importance for biofilm-formation or pathogenicity.

The presence of many bacteria in the biofilm from the CVC not detected by cultivation could potentially be due to DNA from dead bacteria ("naked DNA"), either by natural death or antibiotic treatment, as all the patients included in this study received antibiotics during catheterization. Interestingly, it was found using chinchillas as model system that antibiotic-treated bacteria can persist in a viable state for weeks and can be detected by molecular methods and not cultivation-dependent methods [45], while bacterial DNA from non-viable microorganisms could not be detected by any method. Due to the fact that antibiotics often are used on patients with CVC-related infections cultivation will be hampered and DNA based techniques may be useful in such patient populations [46].

### Microbial populations on internal (luminal) and external (exluminal) site of CVC

When the external and internal site of the catheters were compared the differences in the microbial composition was observed both using DGGE and by comparing the three clone libraries. The clone libaries showed that biofilms from catheters from the external site was dominated by the *Firmicutes*, especially the staphylococci. The second most abundant group was the "uncultured *Deltaproteobacteria*" and they were only found on the external site. The internal site of the CVC was colonized mainly by the *Gammaproteobacteria*. Another study conducted by Donlan [15] supports these observations. It was found that the extent and location of biofilm correlated to the duration of catheterization: short term (< 10 days) catheters had greater biofilm formation on the external surface and long-term catheters (30 days) had more biofilm formation on the catheter inner lumen. Furthermore, some species of the *Gammaproteobacteria*, like *Pseudomonas *sp. appeared to sustain growth in intravenous fluids better than for example the staphylococci [15], which is in accordance with our results.

*Firmicutes *and *Actinobacteria *were often detected using DGGE, both on biofilm from the external and internal site, while the "uncultured *Deltaproteobacteria*" were only found on biofilm from the external site of catheter number 16. This illustrates a preferential PCR amplification of these "uncultured *Deltaproteobacteria*" may have occurred in the clone library. The mixed biofilm sample was dominated in approximately equal extent by the *Firmicutes*, the *Alpha*- and *Betaproteobacteria *and was the only clone library to include *Alphaproteobacteria*. DGGE detected *Alphaproteobacteria *both in internal biofilm from two samples and also in a mixed sample, exemplifying few of the differences obtained by the two molecular approaches.

As Maki's semi-quantitative method was used, the bacteria identified by cultivation must primarily represent bacteria on the external site of the catheters. This was, however, not supported by this study. All bacteria identified by cultivation, except for *K. pneumoniae*, were found by rRNA gene sequencing. This one missing species could either be due to incomplete coverage of the clone libraries or because of loss of material during the rolling of the catheter back and forth on the agar plate prior to the removal of biofilm for the molecular analysis. Importantly, the cultivation approach could have been more intensive in this study, using e.g. sonication to remove cells or anaerobic cultivation techniques, which would probably have resulted in a higher diversity in the detected microorganisms.

### Polymicrobial infections

Using traditional cultivation some reported colonizations on CVC have been polymicrobial, whereas monospecies infections seem to be observed most common [14]. DGGE clearly demonstrated that catheters were in most cases colonized with several species in contrast to the cultivation data primarily showing presence of one or none species. However, in accordance with previous reports, separate bands were observed in some lanes in the DGGE gels representing the same species, for example in sample 1 (external and internal site), sample 6 and 13 (external site) and sample 8 and 11B (internal site) [47-49] and may be due to more than one allele of 16S rRNA genes being present in the same species or presence of different strains of the identified microorganisms with differences in only one or few base pairs. Interestingly, there seemed to be an increased bacterial diversity in biofilm from catheters with a longer insertion time.

### Molecular methods for diagnosis?

Theoretically Maki's semi-quantitative method, the international diagnostic standard [3] primarily detects bacteria on the outer surface of the catheter [3]. However, methods that detect and quantify biofilms both on the inner and the outer surface of catheters will improve detection of biofilm colonizations [15]. Improved methods are also needed for diagnosis while the catheter still is in use [17]. This means that it is essential with an earlier and more precise identification of the pathogens most frequently associated with infections [50,51]. Improvements to the measurement of the efficacy of antimicrobial agents on these bacteria are also required, due to their growth in biofilms [51].

Our observations do not allow us to conclude that all bacteria detected in the biofilm on CVC are important for the biofilm formation or pathogenicity [52]. There is also a potential risk of catheter contamination during removal, even though the clinicians work carefully and aseptically, so we can not completely exclude the possibility that some of the detected microorganisms (using cultivation and molecular methods) did not colonize the catheters in vivo. However, the present study provides a basic list of potential pathogens related to colonizations of CVC, and is a starting point for further investigations. The next step could be to include more catheters in a similar study to increase the available data. Development of a quantitative PCR assay for quantification of the abundance of particular target organisms is also a possibility, e.g. directly on catheter biofilm but also measured in blood samples drawn through the CVC, which could detect the microorganisms on the internal site of the catheters still in situ [6].

## Conclusion

The use of molecular techniques has the potential to substantially improve microbiological diagnosis in CVC-related infections when combined with existing methods based on cultivation and staining.

## Competing interests

The authors declare that they have no competing interests.

## Authors' contributions

MKSL carried out the molecular studies and drafted the manuscript. CM and NH had all patient contact and performed the cultivation analysis. TRT and PHN designed and coordinated the study and helped to draft the manuscript. All authors read and approved the final manuscript.

## Pre-publication history

The pre-publication history for this paper can be accessed here:



## Supplementary Material

Additional file 1 Word format, DGGE data, Identity of bacteria found on the individual catheters, based on sequenced DGGE-bands. X indicates that the species was identified on the catheter. "I" refers to a biofilm sample from the internal site, "E" refers to a biofilm sample from the external site, and "M" is a mixed sample. Accession number for closest relatives are included if they were unknown microorganisms. For catheter number 7 no DGGE data was obtained. Click here for file
