# Supplementary material for: Use of cultivation-dependent and -independent techniques to assess contamination of central venous catheters: a pilot study
Source: BMC Clin Pathol. 2008 Oct 28;8:10. doi: 10.1186/1472-6890-8-10 (PMC2605442; doi:10.1186/1472-6890-8-10)
Supplement: Additional file 1 — Word format, DGGE data, Identity of bacteria found on the individual catheters, based on sequenced DGGE-bands. X indicates that the species was identified on the catheter. "I" refers to a biofilm sample from the internal site, "E" refers to a biofilm sample from the external site, and "M" is a mixed sample. Accession number for closest relatives are included if they were unknown microorganisms. For catheter number 7 no DGGE data was obtained. [file 1472-6890-8-10-S1.doc]

Additional file 1

|  | **Catheter number** |  | 1 |  | 2 |  | 3 |  | 4 |  | 5 |  | 6 |  | 8 |  | 9 |  | | 10 |  | 11 |  | 12 |  | 13 |  | 14 |  | 15 |  | 16 |  | 17 |  | 18 |  |
| --- | --- | --- | --- | --- | --- | --- | --- | --- | --- | --- | --- | --- | --- | --- | --- | --- | --- | --- | --- | --- | --- | --- | --- | --- | --- | --- | --- | --- | --- | --- | --- | --- | --- | --- | --- | --- | --- |
|  | **Species/ Internal or external site** |  | I | E | I | E | I | E | I | E | I | E | I | E | I | E | M | | M | |  | I | E | M |  | I | E | I | E | I | E | I | E | I | E | M |  |
| *Alphaproteobacteria* | *Afipia* sp. MG24 |  |  |  |  |  |  |  |  |  |  |  |  |  |  |  |  |  | |  |  |  |  | X |  |  |  |  |  |  |  |  |  |  |  |  |  |
|  | *Wolbachia* sp. |  |  |  | X |  |  |  |  |  |  |  |  |  |  |  |  |  | |  |  |  |  |  |  |  |  |  |  |  |  |  |  |  |  |  |  |
|  | Uncultured *Mesorhizobium* sp.(AY466731) |  |  |  |  |  | X |  |  |  |  |  |  |  |  |  |  |  | |  |  |  |  |  |  |  |  |  |  |  |  |  |  |  |  |  |  |
| *Betaproteobacteria* | *Delftia acidovorans* |  |  |  |  |  |  | X | X |  |  |  |  |  |  |  | X |  | |  |  |  |  |  |  |  |  |  |  |  |  |  |  |  |  |  |  |
|  | *Diaphorobacter* sp. |  |  |  |  |  |  |  |  |  |  |  |  |  |  |  |  |  | |  |  |  |  |  |  |  |  |  |  |  |  |  |  |  |  | X |  |
|  | *Massilia* sp. |  |  |  |  |  |  |  |  |  |  |  |  | X |  |  |  |  | |  |  |  |  |  |  |  |  |  |  |  |  |  |  |  |  |  |  |
|  | *Oxalobacteraceae* bacterium |  |  |  |  |  |  |  |  |  |  |  |  |  |  |  | X |  | |  |  |  |  |  |  |  |  |  |  |  |  |  |  |  |  |  |  |
| *Gammaproteobacteria* | *Pseudomonas aeruginosa* |  |  |  |  |  |  |  |  |  |  |  |  |  |  |  |  |  | |  |  |  |  |  |  |  |  |  |  |  |  |  |  | X | X |  |  |
|  | *Stenotrophomonas maltophilia* |  |  |  |  | X |  |  |  |  |  |  | X |  |  |  | X |  | |  |  | X |  |  |  |  |  |  |  |  |  |  |  |  |  |  |  |
| *Deltaproteobacteria* | Uncultured *Deltaproteobacterium (*AY222312*)* |  |  |  |  |  |  |  |  |  |  |  |  |  |  |  |  |  | |  |  |  |  |  |  |  |  |  |  |  |  |  | X |  |  |  |  |
| *Firmicutes* | *Enterococcus mundtii* |  |  |  |  |  |  |  |  |  |  |  |  |  |  |  |  |  | |  |  |  | X |  |  |  |  |  |  |  |  |  |  |  |  |  |  |
|  | *Enterococcus sanguinicola* |  |  |  |  |  |  |  |  |  |  |  |  |  |  |  |  |  | |  |  |  | X |  |  |  |  |  |  |  |  |  |  |  |  |  |  |
|  | *Staphylococcus cohnii* |  |  |  |  |  |  |  |  |  |  |  |  |  |  |  |  |  | |  |  |  | X |  |  |  |  |  |  |  |  |  |  |  |  |  |  |
|  | *Staphylococcus epidermidis* |  | X |  | X |  | X |  |  |  |  | X |  |  | X | X |  |  | | X |  |  |  |  |  |  |  | X |  | X | X |  |  |  |  |  |  |
|  | *Staphylococcus haemolyticus* |  |  |  |  |  |  |  |  |  |  |  |  |  |  |  |  |  | |  |  |  |  |  |  |  | X |  |  |  |  |  |  |  |  |  |  |
|  | *Staphylococcus pasteuri* |  |  |  |  |  |  |  |  |  | X |  |  |  |  |  |  |  | |  |  |  |  |  |  |  |  |  |  |  |  |  |  |  |  |  |  |
|  | *Staphylococcus* sp. |  |  |  |  |  |  |  |  |  |  |  |  |  |  |  |  |  | | X |  |  |  |  |  |  |  |  |  |  |  |  |  |  |  |  |  |
|  | Uncultured *Anarococcus* sp.(DQ847450) |  |  |  |  |  |  |  |  |  |  |  |  |  |  |  |  |  | |  |  |  |  |  |  |  |  |  |  |  |  |  |  |  |  | X |  |
|  | Uncultured *Streptoccaceae* bacterium (DQ170578) |  |  |  |  |  |  |  |  |  |  |  |  |  |  |  |  |  | | X |  |  |  |  |  |  |  |  |  |  |  |  |  |  |  |  |  |
| *Actinobacteria* | *Micrococcus luteus* |  |  | X |  |  |  |  |  |  |  |  |  |  |  |  |  |  | |  |  |  |  |  |  |  |  |  |  |  |  |  |  |  |  |  |  |
|  | *Corynebacterium jeikeium* |  |  |  |  |  |  |  |  |  |  |  |  |  |  |  |  |  | |  |  |  |  | X |  |  |  |  |  |  |  |  |  |  |  |  |  |
|  | *Janibacter molonis* |  |  |  |  |  |  | X |  |  |  |  |  |  |  |  |  |  | |  |  |  |  |  |  |  |  |  |  |  |  |  |  |  |  |  |  |
|  | *Propionibacterium acnes* |  |  |  |  | X |  | X | X |  | X |  |  |  |  |  | X |  | |  |  |  |  |  |  | X |  |  |  |  |  |  |  |  |  | X |  |
| *Bacteroidetes* | Uncultured CFB group bacterium (AY038793) |  |  |  |  |  |  |  |  |  |  |  |  |  |  |  |  |  | |  |  |  |  |  |  | X |  |  |  |  |  |  |  |  |  |  |  |
| *Unknown* | Uncultured bacterium mle1-3 (AF280842) |  |  |  |  |  | X |  |  |  |  |  |  |  |  |  |  |  | |  |  |  |  |  |  |  |  |  |  |  |  |  |  |  |  |  |  |
|  | Uncultured bacterium (DQ067035) |  |  |  |  |  |  |  |  |  |  |  |  |  |  |  |  |  | |  |  |  |  |  |  |  |  |  |  |  |  |  |  |  |  | X |  |
